# Morphological Differences in the Abdominal Aorta Between Subjects With and Without Type 2 Diabetes

**DOI:** 10.7759/cureus.68567

**Published:** 2024-09-03

**Authors:** Yuichiro Iwamoto, Tomohiko Kimura, Yukino Katakura, Fuminori Tatsumi, Masashi Shimoda, Shuhei Nakanishi, Tomoatsu Mune, Kohei Kaku, HIdeaki Kaneto

**Affiliations:** 1 Department of Diabetes, Endocrinology, and Metabolism, Kawasaki Medical School, Kurashiki, JPN

**Keywords:** computed tomography, arteriosclerosis, abdominal aortic aneurysm, aorta, type 2 diabetes

## Abstract

Aim

Chronic hyperglycemia is a well-known risk factor for the development of many macrovascular complications, but hyperglycemia may be reportedly protective against abdominal aneurysms.

Materials and methods

In this study, we evaluated morphological differences in the abdominal aorta between subjects with and without type 2 diabetes mellitus (T2DM) without abdominal aortic aneurysm and evaluated the correlation between imaging findings of computed tomography (CT) and diabetes-related parameters.

Results

The abdominal aortic diameter was significantly smaller in subjects with T2DM compared to non-diabetes mellitus (NDM) subjects (p=0.026). Abdominal aortic wall thickness assessed by contrast-enhanced CT was significantly greater in subjects with T2DM compared to NDM subjects (p=0.011). There was a significant single correlation between abdominal aortic diameter and age, gender, Brinkman index, HbA1c, and mean/max intima-media thickness (IMT). Multiple regression analysis showed that HbA1c was an independent negative factor affecting abdominal aortic diameter (t=-3.28, p=0.0036). And Brinkmann index was an independent factor affecting aortic wall thickness (t=2.23, p=0.034).

Conclusion

This study revealed the imaging characteristics of smaller abdominal aortic diameter and larger wall thickness in T2DM subjects compared to NDM subjects. The abdominal aortic wall thickening was significantly correlated with cervical IMT. Therefore, close examination for other diabetes-related macrovascular complications should be aggressively considered when these findings are present.

## Introduction

Type 2 diabetes mellitus (T2DM) causes atherosclerosis in large and small blood vessels due to hyperglycemia and insulin resistance. It is well known that T2DM is a risk factor for cerebral infarction, myocardial infarction, and arteriosclerosis obliterans [1]. The primary pathogenesis of abdominal aortic aneurysm (AAA) is tunica media degeneration of blood vessels. Furthermore, it has been reported recently that the destruction and remodeling of vessel wall structures due to inflammation and immune responses associated with atherosclerosis play an important role in forming dilated lesions [2,3]. In addition, it is reportedly possible that hyperglycemia itself does not exacerbate the risk for abdominal aortic aneurysms [4], which are also caused by atherosclerosis, although it is well-known that hyperglycemia is a risk factor for the development of many other macrovascular complications [5]. Hyperglycemia inhibits the progression of aortic aneurysms by inhibiting neovascularization, macrophage infiltration, and expression of synthetic-type markers in vascular smooth muscle cells [6]. On the other hand, it has been reported that insulin use abolishes this protective effect and accelerates the progression of aortic aneurysms. There is no agreement on whether oral hypoglycemic agents inhibit the progression of aortic aneurysms [4]. The impact of T2DM on the progression of aortic aneurysms is a complex issue that must also consider antidiabetic medications and the degree of glycemic control. Therefore, it is important to evaluate changes in abdominal aortic morphology in patients with T2DM without aortic aneurysm, but this has not been reported. In the present study, we evaluated morphological differences in the abdominal aorta between subjects with and without T2DM without abdominal aortic aneurysm. We assessed the possible correlation of CT findings of the abdominal aorta with patient background, such as age, smoking history, and blood glucose levels, which are associated with diabetic complications.

## Materials and methods

Study population and patient preparation

**Figure 1:**
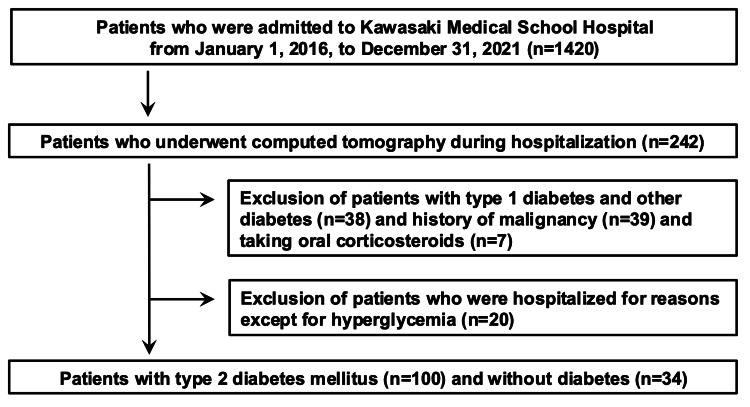
The flowchart of the participants and exclusions in this study.

This retrospective study conducted at the Division of Diabetes, Metabolism, and Endocrinology, Kawasaki Medical School, included adult subjects with T2DM who were admitted to the department and underwent abdominal computed tomography (CT) between January 1, 2016, and December 31, 2021. The study was approved by the Institutional Review Board of Kawasaki Medical School (No. 5686-00). Consent for this study was obtained from patients through an opt-out on the Kawasaki Medical School website. A flowchart of the participants in this study is shown in Figure 1. We first selected 1420 patients admitted to the Division of Diabetes, Metabolism, and Endocrinology, Kawasaki Medical School Hospital, between January 1, 2016, and December 31, 2021. Among them, 242 patients underwent CT during the hospitalization period. We then excluded from the study 38 patients with type 1 diabetes or other diabetes, 39 patients with a history of malignancy, and seven patients taking oral corticosteroids. We then excluded 20 patients with T2DM who were hospitalized for reasons except for hyperglycemia. Finally, we included 100 participants with T2DM and 34 non-diabetic participants in our study. Non-diabetic patients were admitted for the following diseases: nine for adrenal adenomas, nine for electrolyte abnormalities, seven for thyroid dysfunctions, and two each for infection, obesity, and gonadal hormone dysfunction.

Methods

**Figure 2:**
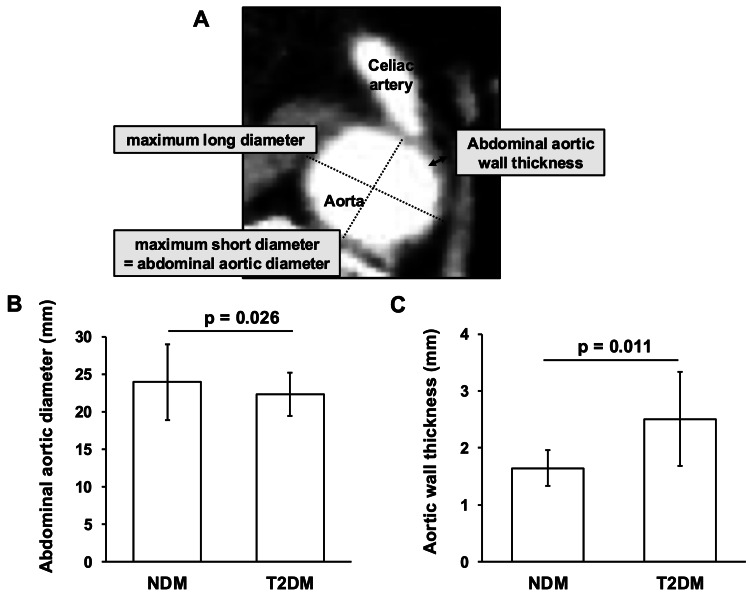
Imaging changes in the abdominal aorta in non-diabetes mellitus (NDM) subjects and type 2 diabetes mellitus (T2DM) subjects. A: Methods for measuring abdominal aortic diameter and abdominal aortic wall thickness; B: Difference in abdominal aortic diameter between NDM and T2DM subjects; C: Difference in abdominal aortic wall thickness between NDM and T2DM subjects NDM: non-diabetes mellitus; T2M: type 2 diabetes mellitus

Morphological differences in the abdominal aorta were assessed using CT image data, which were taken during the hospitalization period. CT examinations were performed using an Aquilion Prime SP (Canon Medical Systems Inc., Tochigi). The measurement method is shown in Figure 2A. The maximum short diameter was defined as the maximum diameter orthogonal to the maximum long diameter, and the measurement site was a short-axis image at the height of the celiac artery bifurcation. In cases where contrast-enhanced CT was performed, the maximum thickness of the aortic wall was also assessed. The relationship between morphological differences in the abdominal aorta and diabetes-related parameters obtained from the medical records was analyzed. Age, gender, Brinkmann index, body weight, body mass index (BMI), systolic blood pressure, and diastolic blood pressure were based on information available at the time of admission. A history of myocardial infarction and cerebral infarction was also interviewed upon admission. HbA1c and glycoalbumin were measured on admission. Early in the morning of the day following admission, blood glucose and C-peptide immunoreactivity (CPR) were measured, and the CPR index was calculated using the following formula: (fasting C-peptide/fasting blood glucose) x 100. Carotid intima-media thickness (IMT) was assessed by an ultrasound technician using ultrasonographic equipment, the Aplio series (CANON MEDICAL SYSTEMS, Tochigi, Japan). The carotid IMT was evaluated in the common carotid artery wall 10 mm centrally from the carotid sinus. The maximum diameter in the same measurement range was defined as max IMT.

Statistical analysis

Data are expressed as mean and standard deviation. The primary endpoint was to evaluate the correlation between imaging changes in the abdominal aorta and diabetes-related parameters. Mann-Whitney's U test and chi-square test were used to compare T2DM and NDM subjects. Spearman's rank correlation coefficient was used to evaluate the correlation between abdominal aortic diameter, aortic wall thickness, and diabetes-related parameters. The carotid intima-media complex (IMT) was evaluated using the natural logarithm for the analysis. Multiple regression analysis was performed using two models to analyze factors affecting abdominal aortic diameter and aortic wall thickness. Model 1 used age, gender, HbA1c, Brinkmann index, and BMI as explanatory variables. In model 2, the explanatory variables were age, sex, HbA1c, Brinkmann index, BMI, systolic blood pressure, serum creatinine, and LDL cholesterol. Parameters related to arterial stiffness were added to the explanatory variables for model 2. JMP (version 16.0.2) was used for data analysis, and Excel for Mac was used to create tables.

## Results

Clinical characteristics of this study participants

The clinical characteristics of the participants in this study are summarized in Table 1. The mean age of all participants was 63.1 ± 15.7 years, and BMI was 25.5 ± 6.9 kg/m2. The mean blood glucose and HbA1c (NGSP) levels in T2DM subjects were 190 ± 97.6 mg/dL and 10.4 ± 2.6%, respectively, which were significantly higher than those in NDM subjects (p<0.0001). T2DM subjects had a larger percentage of males and significantly larger BMI. Systolic blood pressure, Brinkmann index, and LDL cholesterol were 138.8 ± 23.9 mmHg, 371.8 ± 618.2, and 106.6 ± 40.2 mg/dL in T2DM subjects, respectively, compared with 135.5 ± 23.8 mmHg, 172.5 ± 271.9, and 99.7 ± 27.7 mg/dL, respectively, with no significant differences between the two groups.

**Table 1:** Various clinical parameters in this study subjects. Data are presented as mean ± standard deviation. T2DM: type 2 diabetes mellitus; NDM: non-diabetes mellitus; CPR: C-peptide immunoreactivity; BMI: body mass index; LDL: low density lipoprotein; HDL: high density lipoprotein; AST: aspartate aminotransferase; ALT: alanine aminotransferase

Parameters	All subjects (n=134)	T2DM subjects (n=100)	NDM subjects (n=34)	p-value
Male (n, %)	67, 50	57, 57	10, 29	0.0054
Age (years)	63.1 ± 15.7	64.7 ± 13.7	58.2 ± 19.8	0.040
Body weight (kg)	65.7 ± 20.5	68.2 ± 19.5	58.0 ± 21.5	0.013
BMI (kg/m^2^)	25.5 ± 6.9	26.3 ± 6.1	23.1 ± 8.5	0.022
Brinkmann index	325.1 ± 563.1	371.8 ± 618.2	172.5 ± 271.9	n.s
History of myocardial infarction (%)	11.3	14.0	3.0	n.s
History of cerebral infarction (%)	13.5	14.0	12.1	n.s
Systolic blood pressure (mmHg)	138.0 ± 23.9	138.8 ± 23.9	135.5 ± 23.8	n.s
Diastolic blood pressure (mmHg)	80.1 ± 17.9	80.5 ± 17.4	78.9 ± 19.1	n.s
Blood glucose (mg/dL)	179.4 ± 96.7	190.0 ± 97.6	97.6 ± 35.9	<0.0001
HbA1c (%)	9.5 ± 3.0	10.4 ± 2.6	5.6 ± 0.4	<0.0001
Glycoalbumin (%)	28.9 ± 11.5	30.0 ± 11.1	13.2 ± 1.6	0.00045
Serum C-peptide (ng/dL)	2.1 ± 1.2	2.1 ± 1.1	2.5 ± 2.7	n.s
CPR index	1.37 ± 1.09	1.28 ± 0.80	2.56 ± 2.69	0.0024
Triglyceride (mg/dL)	183.0 ± 185.7	192.9 ± 197.6	135.1 ± 98.4	n.s
LDL-cholesterol (mg/dL)	105.4 ± 38.4	106.6 ± 40.2	99.7 ± 27.7	n.s
HDL-cholesterol (mg/dL)	44.0 ± 14.1	43.6 ± 12.7	45.8 ± 19.5	n.s
AST (U/L)	36.1 ± 64.8	30.3 ± 21.6	53.8 ± 122.8	n.s
ALT (U/L)	30.4 ± 32.8	32.2 ± 28.1	24.9 ± 19.8	n.s
Urea nitrogen (mg/dL)	21.2 ± 15.6	22.4 ± 17.0	17.4 ± 9.4	n.s
Creatinine (mg/dL)	0.92 ± 0.66	0.96 ± 0.73	0.80 ± 0.35	n.s

Morphological difference in the abdominal aorta due to type 2 diabetes

Abdominal aortic diameter and abdominal aortic wall thickness in NDM and T2DM subjects are shown in Figure 2. The abdominal aortic diameter in T2DM subjects (22.3 ± 2.9 mm) was significantly smaller compared to NDM subjects (23.9 ± 5.0 mm) (p=0.026, Figure 2B). Abdominal aortic wall thickness in T2DM subjects (2.5 ± 0.8 mm) was significantly greater compared to NDM subjects (1.6 ± 0.3 mm) (p=0.011, Figure 2C).

Correlation of abdominal aortic diameter with various laboratory findings

**Figure 3:**
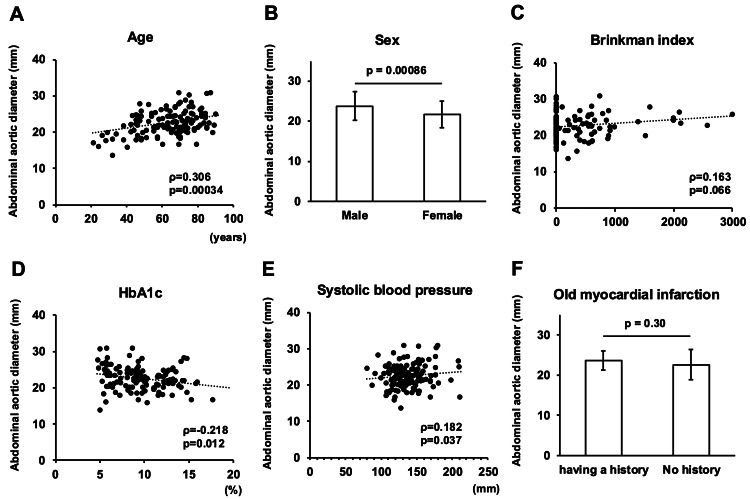
Correlation between abdominal aortic diameter and various clinical parameters. A: age; B: gender; C: Brinkman index; D: HbA1c; E: mean intima-media thickness (IMT); F: max IMT. Correlations were analyzed using Spearman's rank correlation coefficient and Mann-Whitney's U-test.

Table 2 shows the patient background of T2DM subjects divided by median abdominal aortic diameter. Compared to subjects with smaller abdominal aortic diameters, those with larger diameters were more often male (p=0.026), older (p=0.0041), and had lower HbA1c (p=0.0046) and higher Brinkmann index (p=0.0091). Figure 3 shows various parameters that were correlated with abdominal aortic diameter in all subjects. The abdominal aortic diameter was significantly larger in males (23.8 ± 3.6 mm) compared to females (21.7 ± 3.3 mm) (p=0.00086). A significant positive correlation was found between abdominal aortic diameter and age (ρ=0.306, p=0.00034). There was a significant negative correlation between abdominal aortic diameter and HbA1c (ρ=-0.218, p=0.012). Brinkmann index was not an independent factor affecting abdominal aortic diameter (ρ=0.163, p=n.s.). Significant positive correlations were found between abdominal aortic diameter and mean IMT and max IMT (ρ=0.263, p=0.034, ρ=0.323, p=0.00020). Next, to evaluate possible factors independently affecting abdominal aortic diameter, we performed multiple regression analyses. As shown in Table 3, in model 1, HbA1c and gender were independent negative factors affecting abdominal aortic diameter (t=-2.97, p=0.036; t=-4.07, p<0.0001). Age also positively affected abdominal aortic diameter (t=4.11, p<0.0001). Similar results were obtained in model 2, which included other arterial stiffness factors. HbA1c and gender were independent negative factors affecting abdominal aortic diameter (t=-2.88, p=0.0048; t=-4.00, p=0.00012). Age and systolic blood pressure were positive factors affecting abdominal aortic diameter (t=3.61, p=0.00047; t=2.15, p=0.034).

**Table 2:** Differences in diabetes-related parameters when T2DM subjects are grouped by aortic diameter. Data are presented as mean ± standard deviation. T2DM: type 2 diabetes mellitus; BMI: body mass index; IMT: intima-media complex; ABI: ankle brachial index; CAVI: cardio-ankle vascular index *: p < 0.05 with chi-square test and analysis of variance (ANOVA)

Parameters	T2DM subjects with aortic diameter greater than median (n=50)	T2DM subjects with aortic system smaller than median (n=50)	p-value
Male / female	34 / 16	23 / 27	0.026
Age (years)	68.6 ± 11.0	60.9 ± 15.2	0.0041
Body weight (kg)	70.5 ± 19.7	66.0 ± 19.5	0.25
BMI (kg/m^2^)	27.0 ± 6.4	25.6 ± 5.8	0.26
Brinkmann index	533.9 ± 781.0	209.7 ± 341.7	0.0091
Duration of T2DM	15.8 ± 13.4	13.2 ± 10.1	0.27
History of myocardial infarction (%)	22.0	6.0	0.021
History of cerebral infarction (%)	22.0	6.0	0.021
Blood glucose (mg/dL)	174.4 ± 57.0	205.9 ± 125.8	0.11
HbA1c (%)	9.7 ± 2.4	11.1 ± 2.6	0.0046
Glycoalbumin (%)	27.2 ± 9.7	32.7 ± 12.0	0.020
Diabetic neuropathy progressing from Baba’s Neuropathy Classification 1(%)	40.5	52.6	0.29
Diabetic retinopathy (%)	28.3	31.8	0.72
Diabetic nephropathy stage 2 or more advanced (%)	42.9	35.4	0.45
Mean IMT (mm)	0.98 ± 0.81	0.81 ± 0.22	0.016
Max IMT (mm)	1.35 ± 0.62	1.01 ± 0.29	0.0029
ABI	1.06 ± 0.13	1.08 ± 0.23	0.72
CAVI	9.3 ± 1.7	8.5 ± 1.3	0.030
Drugs used during hospitalization			
Insulin (%)	26.0	26.0	1.00
GLP-1 receptor agonist (%)	6.0	14.0	0.18
DPP4 inhibitor (%)	54.0	46.0	0.42
Sulfonylurea or glynide (%)	28.0	36.0	0.39
Biganide (%)	40.0	34.0	0.53
Pioglitazone (%)	10.0	16.0	0.37
Alpha glucosidase inhibitor (%)	10.0	16.0	0.37
SGLT2 inhibitor (%)	18.0	22.0	0.62

**Table 3:** Multiple regression analysis to evaluate factors independently affecting abdominal aortic diameter.

Model 1	t-value	p-value
Age (years)	4.11	<0.0001
Female	-4.07	<0.0001
HbA1c (%)	-2.97	0.0036
Brinkman index	0.7	n.s
Body mass index (kg/m^2^)	0.79	n.s
Model 2	t-value	p-value
Age (years)	4.40	<0.0001
Female	-4.01	0.0001
HbA1c (%)	-0.86	n.s
Brinkman index	0.43	n.s
Body mass index (kg/m^2^)	2.63	0.011
History of myocardial infarction	0.62	n.s
Systolic blood pressure (mmHg)	0.52	n.s
Cardio-ankle vascular index	-0.85	n.s

Correlation of abdominal aortic wall thickness with various laboratory findings

**Figure 4:**
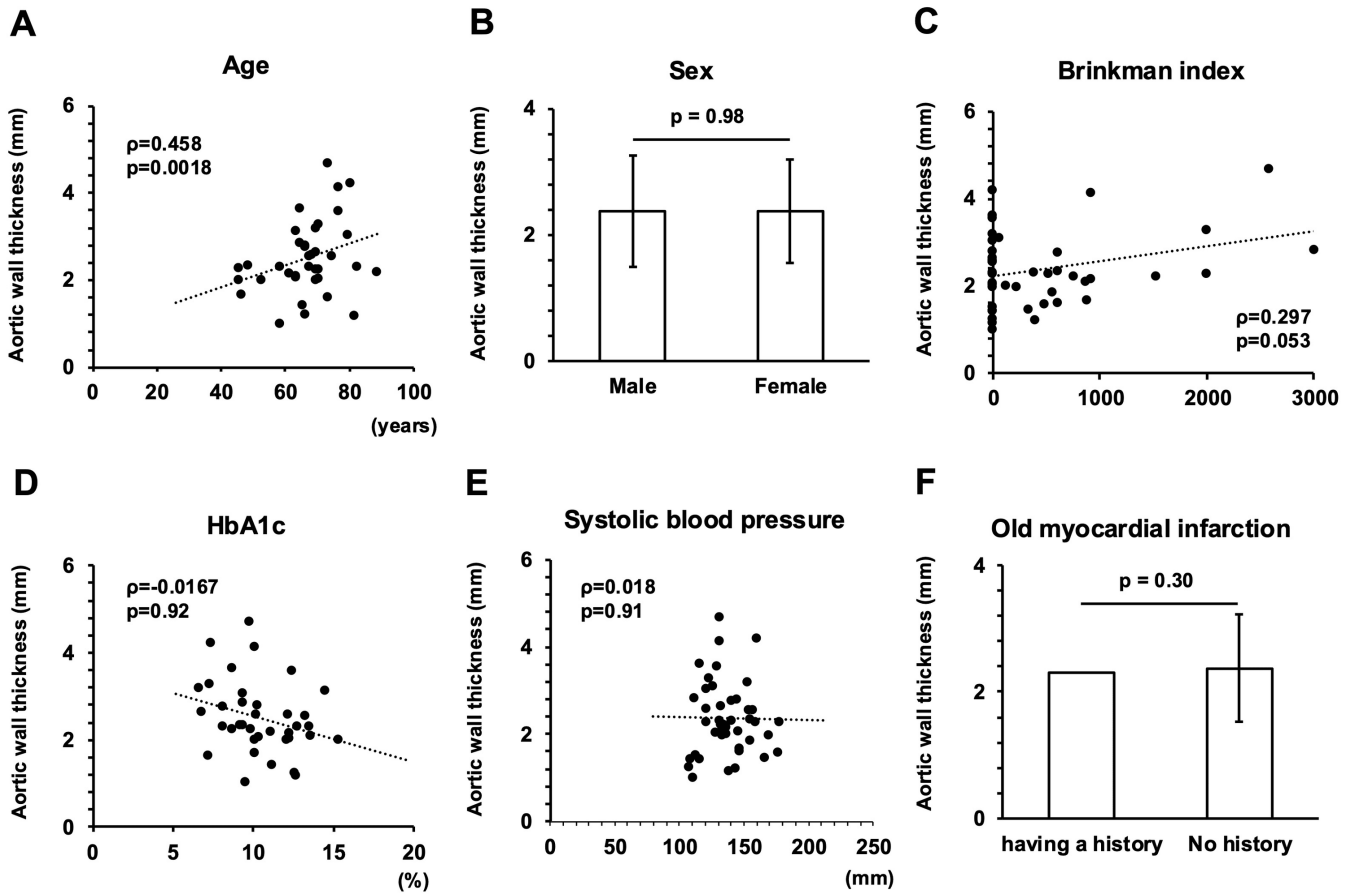
Correlation between abdominal aortic wall thickness and various clinical parameters. A: age; B: gender; C: Brinkman index; D: HbA1c; E: mean intima-media thickness (IMT); F: max IMT. Correlations were analyzed using Spearman's rank correlation coefficient and Mann-Whitney's U-test.

Figure 4 shows various parameters that were correlated with abdominal aortic wall thickness in 44 patients who underwent contrast-enhanced CT. Abdominal aortic wall thickness was positively correlated with age, Brinkmann index, and CPR index (ρ=0.305, p<0.0001; ρ=0.262, p=0.00051; ρ=0.203, p=0.019). A significant negative correlation was found between aortic wall thickness and HbA1c (r=-0.285, p=0.00087). Mean IMT and max IMT were positively correlated with abdominal aortic wall thickness (ρ=0.657, p<0.0001, ρ=0.597, p<0.0001).

Next, to assess possible factors independently affecting abdominal aortic wall thickness, we performed multiple regression analyses. As shown in Table 4, HbA1c was not an independent factor affecting aortic wall thickness in model 1. Age and Brinkmann index were independent positive factors affecting aortic wall thickness (t=3.10, p=0.0038; t=2.25, p=0.031 in model 1).

**Table 4:** Multiple regression analysis to evaluate factors independently affecting abdominal aortic wall thickness.

Model 1	t-value	p-value
Age (years)	4.11	<0.0001
Female	-4.07	<0.0001
HbA1c (%)	-2.97	0.0036
Brinkman index	0.7	n.s
Body mass index (kg/m^2^)	0.79	n.s
Model 2	t-value	p-value
Age (years)	4.40	<0.0001
Female	-4.01	0.0001
HbA1c (%)	-0.86	n.s
Brinkman index	0.43	n.s
Body mass index (kg/m^2^)	2.63	0.011
History of myocardial infarction	0.62	n.s
Systolic blood pressure (mmHg)	0.52	n.s
Cardio-ankle vascular index	-0.85	n.s

These results showed that both abdominal aortic diameter and abdominal aortic wall thickness were mono-correlated with HbA1c. And a new finding in this study is that in multiple regression analysis with the addition of factors related to atherosclerosis, HbA1c was an independent factor affecting abdominal aortic diameter. In contrast, abdominal aortic wall thickness was an independent factor affecting the other factors, particularly strongly influenced by smoking history.

## Discussion

In the present study, we investigated the changes in maximum short diameter and wall thickness of the abdominal aorta in T2DM subjects compared to NDM subjects without aortic aneurysms. It is possible that chronic hyperglycemia reduced aortic diameter expansion in subjects without aortic aneurysms. On the other hand, it was suggested that the thickening of the aortic wall may be accompanied by the progression of other macrovascular complications. These results provide an important indicator for evaluating macrovascular complications in T2DM subjects.

Previous studies of patients with abdominal aortic aneurysm (AAA) showed that there was a negative correlation between blood glucose levels and the rate of aneurysmal diameter expansion and that hyperglycemia is risk-reducing for AAA [4]. The risk-reducing effects of hyperglycemia on AAA include reduction of aortic wall neovascularization [7], aortic IMT thickening [8], collagen resistance to collagen degradation [9], and apoptosis of aortic smooth muscle cells [10]. In the present study, there was a negative correlation between HbA1c and aortic diameter in T2DM subjects without AAA, which was compatible with the previous studies. Therefore, it is possible that chronic hyperglycemia inhibits tunica media degeneration of the aortic wall even before the formation of aortic aneurysms. Both abdominal aortic diameter and abdominal aortic wall thickness were correlated with similar items, such as age, smoking, and HbA1c, but multiple regression analysis revealed different trends in independent influencing factors. Further studies are warranted because these findings may reflect different pathological conditions, such as arterial stiffness and atherosclerosis.

The risk of AAA with smoking is 4.87 times (95%CI: 3.93-6.02) [11]. Nicotine in cigarettes is believed to contribute to the development of AAA by accelerating the degradation of collagen fibers and increasing oxidative stress on blood vessels [12]. In the present study, the Brinkmann index was an independent factor for abdominal aortic wall thickening in subjects without AAA. The possible reason for this is as follows. First, resistance to collagen degradation due to hyperglycemia may be abolished by smoking. Second, resistance to collagen degradation may be abolished in T2DM subjects with vascular complications. Third, both diabetes and smoking may have reciprocally increased oxidative stress in the vascular wall. Smoking history was not a factor affecting abdominal aortic diameter in this study. A history of smoking for more than 20 pack years has been reported to increase the risk of abdominal aortic aneurysm enlargement in women [13]. The mean Brinkmann index of the participants in this study was 325.1 ± 563.1, which may be influenced by the small number of smokers over 20 pack years and the sample size of the study.

There are several limitations in this study. First, this is a single-center, retrospective observational study. Second, the mean HbA1c of the T2DM participants in this study was markedly high (10.4 ± 2.6%), and the patient background may differ from that of T2DM subjects in a non-specialty center. Second, although it has been shown that hyperglycemia may affect aortic wall morphology, it is difficult to determine the mechanism or the effect of antidiabetic drugs. Next, the participants without diabetes are patients who were hospitalized for testing or treatment of other diseases, and there may be a different group of participants than healthy adults without any disease. And since the patient backgrounds of T2DM subjects and non-DM subjects are very different, we cannot rule out the possibility that factors other than blood glucose levels may have influenced the results. Further studies with more patients and more confounding factors are warranted. Finally, this study used CT to assess aortic morphology, but the imaging method was not synchronized with the heartbeat, which raises concerns about bias due to the timing of imaging. For a more accurate evaluation, it is desirable to use a heartbeat-synchronized imaging method or an ultrasound device in the future.

## Conclusions

In conclusion, this study revealed the imaging characteristics of smaller abdominal aortic diameter and larger wall thickness in T2DM subjects compared to NDM subjects. This study also shows that HbA1c may reduce the risk of aortic diameter enlargement even in subjects without AAA. On the other hand, the abdominal aortic wall was thicker in T2DM subjects, and aortic wall thickness was significantly correlated with cervical IMT. Therefore, a close examination for other diabetes-related macrovascular complications should be aggressively considered when these findings are present.
